# Supplementary material for: Regulatory T cells induce polarization of pro-repair macrophages by secreting sFGL2 into the endometriotic milieu
Source: Commun Biol. 2021 Apr 23;4:499. doi: 10.1038/s42003-021-02018-z (PMC8065041; doi:10.1038/s42003-021-02018-z)
Supplement: Supplementary file 2 — Supplementary Information [file 42003_2021_2018_MOESM2_ESM.pdf]

Supplementary Fig. 1 Morphological changes in THP-1 and U937 cells macrophages differentiation.

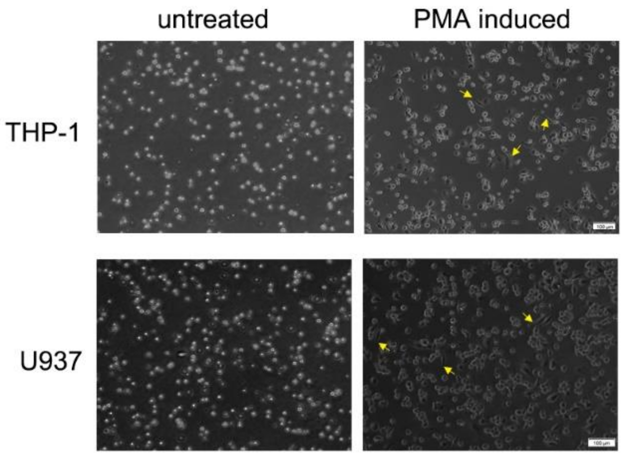

Representative images of untreated THP-1 and U937 cells and cells in the presence of 100 ng per ml PMA for 48 h. Yellow arrows denote pseudopodia.

Supplemental Fig. 2 FGL2 had no effect on P38, JNK and STAT6 phosphorylation in THP-1 and U937 cells.

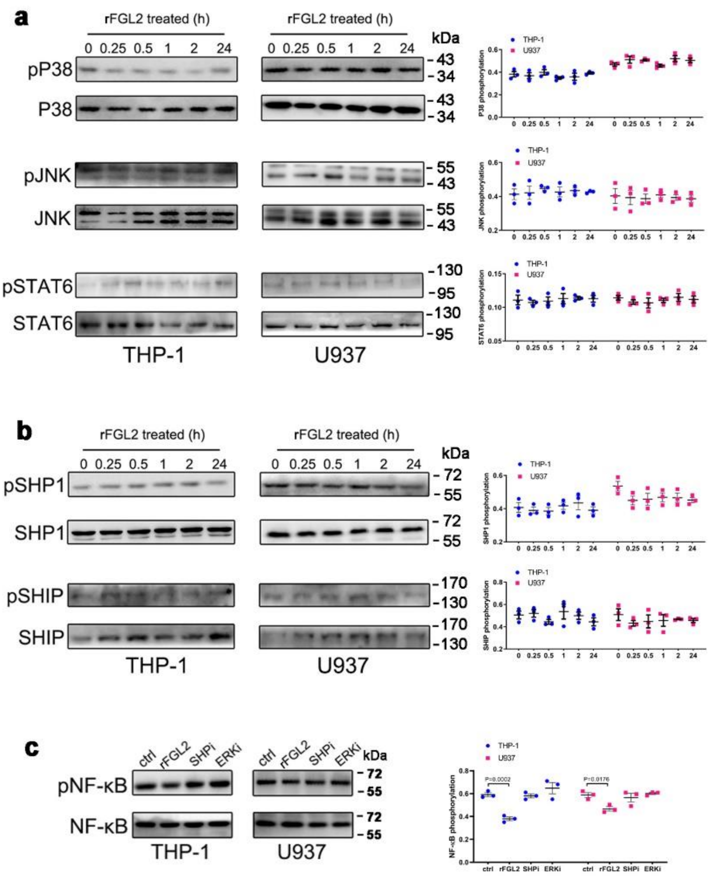

The phosphorylation of P38, JNK, STAT6, SHP1, SHIP and NF-κB was detected by western blot. Data represent the mean value ± SEM of three individual experiments (n=3).

Supplemental Fig. 3 Cell transfection efficiency in THP-1 and U937 cells.

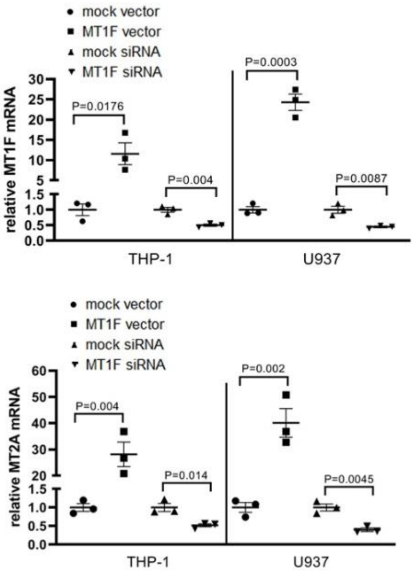

The transfection efficiency of MT1F and MT2A in THP-1 and U937 cells was analyzed by qRT-PCR. Data represent the mean value ± SEM of three individual experiments (n=3).

Supplemental Fig. 4 The expression of CD32B on NK and T cells in tissues.

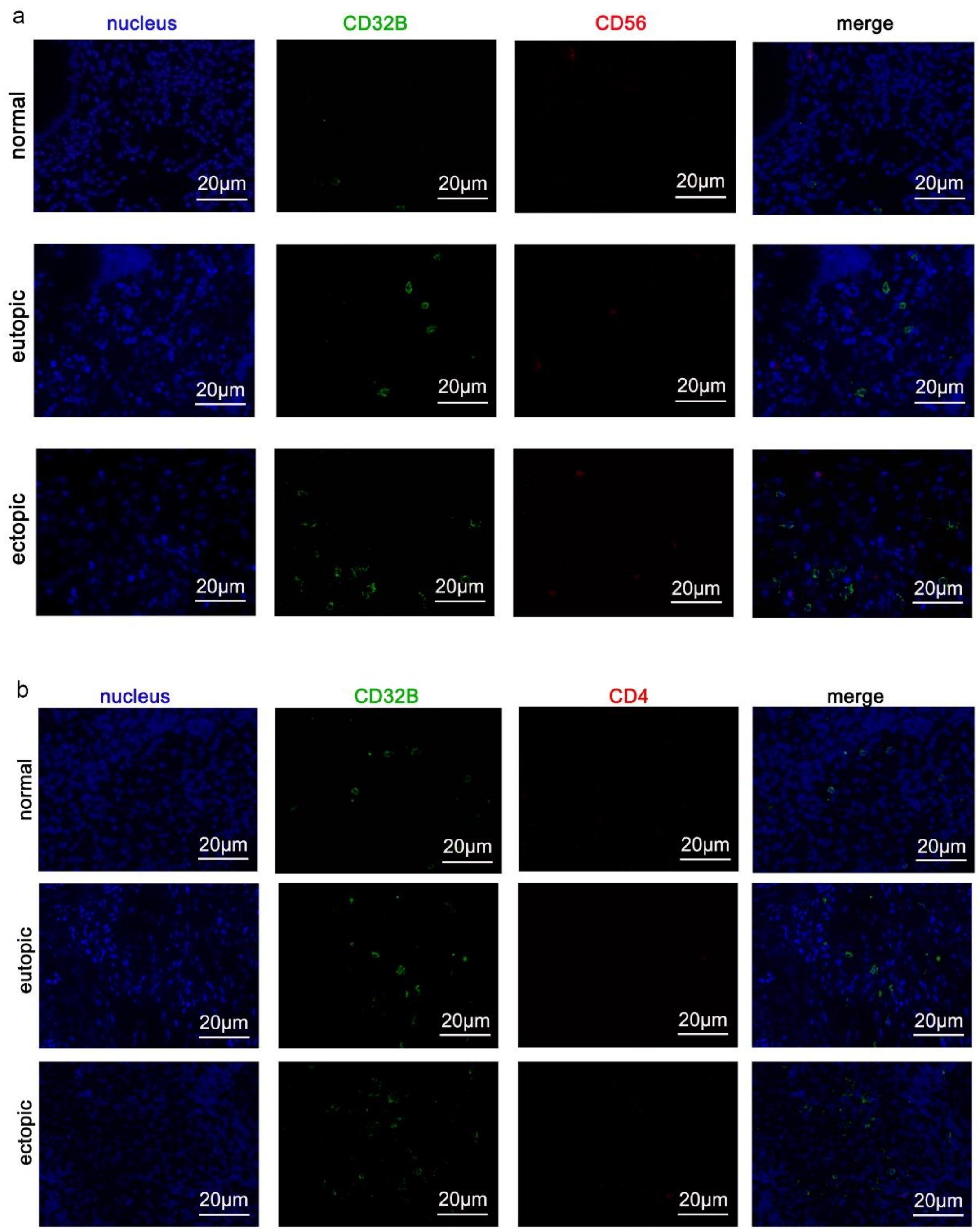

(a) Confocal microscopic analysis of nucleus (blue), CD32B (green), NCAM1 (CD56, RNL-1, red), expression in normal endometrium, eutopic

endometrium and ectopic lesions. **(b)** Confocal microscopic analysis of nucleus (blue), CD32B (green), CD4 (red), expression in normal endometrium, eutopic endometrium and ectopic lesions. A few CD32B positive cells were NK and T cells.

Supplementary Table 1. Key resources table.

REAGENT RESOURCE	SOURCE	IDENTIFIER
Antibodies		
APC-Anti-human CD86	Biolegend	Cat# 374208
Percp cy5.5-Anti-human HLA-DR	Biolegend	Cat# 307630
PE-Anti-human IL-6	Biolegend	Cat# 501107
647-Anti-human CD163	Biolegend	Cat# 326508
FITC-Anti-human CD206	Biolegend	Cat# 321104
APC-Anti-human TGF-β	Biolegend	Cat# 349608
FITC-donkey anti-rabbit IgG	Biolegend	Cat# 406403
Rabbit anti-human FGL2	Abcam	Cat# ab198029
Rabbit anti-human CD32B	Abcam	Cat# ab45143
Goat anti-human CD32B	Abcam	Cat# 110076
Mouse anti-CD68 antibody	Abcam	Cat# ab955
Mouse anti-human NCAM1 (RNL-1, CD56)	Abcam	Cat# ab9018
Mouse anti-CD4 antibody	R&D	Cat# MAB379
donkey anti-goat IgG (Alexa Fluor 488)	Abcam	Cat# 150129
donkey anti-mouse IgG (Alexa Fluor 647)	Abcam	Cat# 150107
Goat anti-rabbit IgG (Alexa Fluor 488)	Abcam	Cat# ab181448
Goat anti-mouse IgG (Alexa Fluor 647)	Abcam	Cat# ab150119
Anti-human pNF-κB	Cell Signaling	Cat# 3033
Anti-human NF-κB	Cell Signaling	Cat# 8242
Anti-human pERK1/2	Cell Signaling	Cat# 4370
Anti-human ERK1/2	Cell Signaling	Cat# 4695
Anti-human pSTAT3	Cell Signaling	Cat# 9138
Anti-human STAT3	Cell Signaling	Cat# 9139
Anti-human pSHP2	Cell Signaling	Cat# 3751
Anti-human SHP2	Cell Signaling	Cat# 3397
Anti-human pSHIP	Cell Signaling	Cat# 2725

Anti-human SHIP	Cell Signaling	Cat# 3941
Anti-human pSHP1	Bioworld	Cat# BS94059
Anti-human SHP1	Bioworld	Cat# BS91238
anti-Mouse second antibodies	Bioworld	Cat# BS12478
anti-Rabbit second antibodies	Bioworld	Cat# BS10043
Chemicals, Peptides, and Recombinant Proteins		
Recombinant Human M-CSF	Peprotech	Cat# AF-300-25
Recombinant Human IFN-γ	Peprotech	Cat# AF-300-02
Recombinant Human IL-4	Peprotech	Cat# AF-200-04
Recombinant Human FGL2	Abnova	Cat# H00010875-P01
SHP2 inhibitor	Millipore	Cat# 565851
ERK inhibitor	Selleck	Cat# SCH772984
STAT3 inhibitor	Selleck	Cat# S3I-201
Ficoll	Sigma-Aldrich	Cat# F2637
PBS	GIBCO	Cat# 20012-068
Fetal bovine serum	HyClone	Cat# SH30406.05
DMEM/F12	HyClone	Cat# SH30023.01
RPMI 1640 Medium	GIBCO	Cat# 21875034
type IV collagenase	Sigma	Cat# M9445
LPS	Sigma	Cat# L2630
DAPI	Cell Signaling	Cat# 4083
PMA	Sigma	Cat# P8139
ionomycin	Sigma	Cat# I3909
Monensin	BD Biosciences	Cat# M5273
Lipofectamine 2000	Invitrogen	Cat# 11668-019
ECL	Millipore	Cat# WBKLS01 00
Critical Commercial Assays		
Human Gene Expression Array	Affymetrix	Cat# GSE133867
Mo cell Isolation Kit	Miltenyi Biotec	Cat# 130-050-201
Treg cell Isolation Kit	Miltenyi Biotec	Cat# 130-091-301
Tregs expansion kit	Miltenyi Biotec	Cat# 130-095-353
NK cell Isolation Kit	Miltenyi Biotec	Cat# 130-092-657
naïve CD4+ T cell isolation kit	Miltenyi Biotec	Cat# 130-094-131
qPCR Mix Kit	Takara	Cat# RR037A
human FGL2 ELISA kits	BioLegend	Cat# 436907
human IL-10 ELISA kits	R&D	Cat# D1000B
human IL-12p70 ELISA kits	R&D	Cat# D1200
Fix & Perm cell permeabilization kit	BioLegend	Cat# 421401
BCA protein assay	Beyotime	Cat# P0010
Deposited Data		
Experimental Models: Cell Lines		
THP-1	ATCC	Cat# ATCC® TIB-202™
U937	ATCC	Cat# ATCC® CRL-1593.2™
Oligonucleotides		

Primer: T-bet (FWD): TGGAGGTGTCGGGGAAACT (5'-3')	This paper	N/A
Primer: T-bet (REV): TGGGAACATCCGCCGT (5'-3')	This paper	N/A
Primer: GATA3 (FWD): TGTGCTCGGAGGGTTTCTTG (5'-3')	This paper	N/A
Primer: GATA3 (REV): GCACGCTGGTAGCTCATACA (5'-3')	This paper	N/A
Primer: RORC (FWD): CTGAGAAGGACAGGGAGCCA (5'-3')	This paper	N/A
Primer: RORC (REV): CGTAGTGGATCCCAGACGAC (5'-3')	This paper	N/A
Primer: FOXP3 (FWD): TCCCAGAGTTCCTCCACAAC (5'-3')	This paper	N/A
Primer: FOXP3 (REV): ATTGAGTGTCCGCTGCTTCT (5'-3')	This paper	N/A
Primer: FGL2 (FWD): TCCCAGAGTTCCTCCACAAC (5'-3')	This paper	N/A
Primer: FGL2 (REV): GGTGAAGTTGGTGCTCCCAT (5'-3')	This paper	N/A
Primer: NF-κB (FWD): TTTGGGAAGGCCTGAACAAATG (5'-3')	This paper	N/A
Primer: NF-κB (REV): GGTATGGGCCATCTGCTGTT (5'-3')	This paper	N/A
Primer: STAT1 (FWD): GTTATGGGACCGCACCTTCA (5'-3')	This paper	N/A
Primer: STAT1 (REV): CAGTGAACTGGACCCCTGTC (5'-3')	This paper	N/A
Primer: C/EBPα (FWD): GGTGGACAAGAACAGCAACGA (5'-3')	This paper	N/A
Primer: C/EBPα (REV): GTCATTGTCACTGGTCAGCTC (5'-3')	This paper	N/A
Primer: MT1E (FWD): ATGGACCCCAACTGCTCTT (5'-3')	This paper	N/A
Primer: MT1E (REV): CTTCTCCGATGCCCCTTT (5'-3')	This paper	N/A
Primer: MT1F (FWD): ATGGACCCCAACTGCTCCT (5'-3')	This paper	N/A
Primer: MT1F (REV): ACTTCTCTGACGCCCCTTTG (5'-3')	This paper	N/A
Primer: MT1G (FWD): ATGGACCCCAACTGCTCCT (5'-3')	This paper	N/A
Primer: MT1G (REV): ACTTCTCCGATGCCCCTTT (5'-3')	This paper	N/A

Primer: MT1M (FWD): GCAAGTGCAAAGAGTGCAA (5'-3')	This paper	N/A
Primer: MT1M (REV): CAGTTCTCCAACGTCCCTTT (5'-3')	This paper	N/A
Primer: MT2A (FWD): ATGGATCCCAACTGCTCCT (5'-3')	This paper	N/A
Primer: MT2A (REV): GCAGCAGCTTTTCTTGCAG (5'-3')	This paper	N/A
Primer: NOS2 (FWD): ATTCACTCAGCTGTGCATCG (5'-3')	This paper	N/A
Primer: NOS2 (REV): TCAGGTGGGATTTCGAAGAG (5'-3')	This paper	N/A
Primer: Arg1 (FWD): CTCAAGCAGACCAGCCTTTC (5'-3')	This paper	N/A
Primer: Arg1 (REV): GCCAAGTCCAGAACCATAGG (5'-3')	This paper	N/A
Negative control siRNA:<br>sense (5'-3'):<br>UUC UCC GAA CGU GUC ACG UTT<br>antisense (5'-3'):<br>ACG UGA CAC GUU CGG AGA ATT	GenePharma	N/A
MT1F siRNA:<br>Sense (5'-3'):<br>GAC AUG UAC AAA CCU GGA UTT<br>Antisense (5'-3'):<br>AUC CAG GUU UGU ACA UGU CTT	GenePharma	N/A
MT2A siRNA:<br>Sense (5'-3'):<br>CGC UCC CAG AUG UAA AGA ATT<br>Antisense (5'-3'):<br>UUC UUU ACA UCU GGG AGC GTT	GenePharma	N/A
Software and Algorithms		
Prism v.8.0	GraphPad	http://www.graphpad.com
