# Supplementary material for: Regulatory T cells induce polarization of pro-repair macrophages by secreting sFGL2 into the endometriotic milieu
Source: Commun Biol. 2021 Apr 23;4:499. doi: 10.1038/s42003-021-02018-z (PMC8065041; doi:10.1038/s42003-021-02018-z)
Supplement: Supplementary file 5 — Reporting Summary [file 42003_2021_2018_MOESM5_ESM.pdf]

nature research

Corresponding author(s): DBPR COMMSBIO-20-2122-T

Last updated by author(s): Feb 3, 2021

Reporting Summary

Nature Research wishes to improve the reproducibility of the work that we publish. This form provides structure for consistency and transparency in reporting. For further information on Nature Research policies, see our Editorial Policies and the Editorial Policy Checklist.

Statistics

For all statistical analyses, confirm that the following items are present in the figure legend, table legend, main text, or Methods section.

n/a	Confirmed	
☐	☒	The exact sample size (*n*) for each experimental group/condition, given as a discrete number and unit of measurement
☐	☒	A statement on whether measurements were taken from distinct samples or whether the same sample was measured repeatedly
☐	☒	The statistical test(s) used AND whether they are one- or two-sided<br>*Only common tests should be described solely by name; describe more complex techniques in the Methods section.*
☒	☐	A description of all covariates tested
☐	☒	A description of any assumptions or corrections, such as tests of normality and adjustment for multiple comparisons
☐	☒	A full description of the statistical parameters including central tendency (e.g. means) or other basic estimates (e.g. regression coefficient) AND variation (e.g. standard deviation) or associated estimates of uncertainty (e.g. confidence intervals)
☐	☒	For null hypothesis testing, the test statistic (e.g. *F*, *t*, *r*) with confidence intervals, effect sizes, degrees of freedom and *P* value noted<br>*Give P values as exact values whenever suitable.*
☒	☐	For Bayesian analysis, information on the choice of priors and Markov chain Monte Carlo settings
☒	☐	For hierarchical and complex designs, identification of the appropriate level for tests and full reporting of outcomes
☒	☐	Estimates of effect sizes (e.g. Cohen's *d*, Pearson's *r*), indicating how they were calculated

Our web collection on statistics for biologists contains articles on many of the points above.

Software and code

Policy information about availability of computer code

Data collection	Gene Expression Array data GEO: GSE133867
Data analysis	GraphPad Prism 8.0

For manuscripts utilizing custom algorithms or software that are central to the research but not yet described in published literature, software must be made available to editors and reviewers. We strongly encourage code deposition in a community repository (e.g. GitHub). See the Nature Research guidelines for submitting code & software for further information.

Data

Policy information about availability of data

All manuscripts must include a data availability statement. This statement should provide the following information, where applicable:

- Accession codes, unique identifiers, or web links for publicly available datasets
- A list of figures that have associated raw data
- A description of any restrictions on data availability

The source data that support the findings of this study are available from the corresponding author upon request. The accession number for the Gene Expression Array data reported in this paper is GEO: GSE133867. All other data needed to evaluate the conclusions in the paper are present in the paper or the Supplementary Materials.

Field-specific reporting

Please select the one below that is the best fit for your research. If you are not sure, read the appropriate sections before making your selection.

[x] Life sciences [ ] Behavioural & social sciences [ ] Ecological, evolutionary & environmental sciences

For a reference copy of the document with all sections, see nature.com/documents/nr-reporting-summary-flat.pdf

Life sciences study design

All studies must disclose on these points even when the disclosure is negative.

Sample size	In this study,48 endometriosis patients were surgically staged, including 27 in stage I-II, and 21 in stage III-IV. Normal endometrial tissues were collected from 20 age-matched patients. Controlled peritoneal fluid was collected from 34 women who underwent laparoscopy for cystic teratoma or hysteromyomectomy. According to the previous published articles and results, these samples were sufficient for the one-way ANOVA analysis and t test.
Data exclusions	No data were excluded from the analyses.
Replication	We repeated our experiment for at least three times with similar results.
Randomization	This is not relevant to our study. Our participants were not involved in experimental groups.
Blinding	This is not relevant to our study. Our participants were not involved in experimental groups.

Reporting for specific materials, systems and methods

We require information from authors about some types of materials, experimental systems and methods used in many studies. Here, indicate whether each material, system or method listed is relevant to your study. If you are not sure if a list item applies to your research, read the appropriate section before selecting a response.

Materials & experimental systems

n/a	Involved in the study	
[ ]	[x]	Antibodies
[ ]	[x]	Eukaryotic cell lines
[x]	[ ]	Palaeontology and archaeology
[x]	[ ]	Animals and other organisms
[ ]	[x]	Human research participants
[x]	[ ]	Clinical data
[x]	[ ]	Dual use research of concern

Methods

n/a	Involved in the study	
[x]	[ ]	ChIP-seq
[ ]	[x]	Flow cytometry
[x]	[ ]	MRI-based neuroimaging

Antibodies

Antibodies used	Data provided in the manuscript.
Validation	Data provided in the manuscript.

Eukaryotic cell lines

Policy information about cell lines

Cell line source(s)	THP-1 and U937 monocytic cell lines were purchased from ATCC.
Authentication	Cell Line Authentication by STR profiling.
Mycoplasma contamination	The cell lines were not tested for mycoplasma contamination.
Commonly misidentified lines (See ICLAC register)	None.

Human research participants

Policy information about studies involving human research participants

Population characteristics	The paticipants were all Chinese female, diagnosis were endometriosis (N80.000).
Recruitment	Before surgery, we explained our study to them, and informed consent was obtained from all subjects.
Ethics oversight	Ethics Committees of Obstetrics and Gynecology Hospital of Fudan University

Note that full information on the approval of the study protocol must also be provided in the manuscript.

Flow Cytometry

Plots

Confirm that:

- [x] The axis labels state the marker and fluorochrome used (e.g. CD4-FITC).
- [x] The axis scales are clearly visible. Include numbers along axes only for bottom left plot of group (a 'group' is an analysis of identical markers).
- [x] All plots are contour plots with outliers or pseudocolor plots.
- [x] A numerical value for number of cells or percentage (with statistics) is provided.

Methodology

Sample preparation	Cells were stained with antibodies to cell surface antigens for 30 min in the dark on ice. For intracellular cytokine staining, the cells were stimulated with PMA (50 ng per ml, Sigma, USA), ionomycin (1 μg per ml, Sigma, USA) and monensin (1 μl per ml, BD Biosciences, USA) 4 h before testing. Intracellular staining procedure was carried out according to the manufacturer's instructions with the Fix & Perm cell permeabilization kit (BioLegend, CA, USA).
Instrument	Flow cytometry was performed on a CyAn ADP machine (Beckman coulter, USA).
Software	Data were analyzed with Flowjo software (Tree Star)
Cell population abundance	PBMC were isolated from heparinized peripheral venous blood samples via Ficoll density gradient centrifugation. CD14 MicroBeads (Miltenyi Biotec, Germany) were used for the magnetic positive selection of human CD14+ monocyte from PBMC, and then were determined using Flow cytometry.
Gating strategy	By setting and adjusting FSC/SSC, most of the interference of cell debris, bubbles and noise is excluded from the analysis area. Use a suitable fluorescent negative control, and exclude dead cells. Circling the cell population of interest in the dot plot.

- [ ] Tick this box to confirm that a figure exemplifying the gating strategy is provided in the Supplementary Information.
